# mRMR-ABC: A Hybrid Gene Selection Algorithm for Cancer Classification Using Microarray Gene Expression Profiling

**DOI:** 10.1155/2015/604910

**Published:** 2015-04-15

**Authors:** Hala Alshamlan, Ghada Badr, Yousef Alohali

**Affiliations:** ^1^College of Computer and Information Sciences, King Saud University, P.O. Box 22452, Riyadh 11495, Saudi Arabia; ^2^IRI, The city for Science and Technology, University and Research District, P.O. Box 21934, New Borg Al-Arab, Alexandria, Egypt

## Abstract

An artificial bee colony (ABC) is a relatively recent swarm intelligence optimization approach. In this paper, we propose the first attempt at applying ABC algorithm in analyzing a microarray gene expression profile. In addition, we propose an innovative feature selection algorithm, minimum redundancy maximum relevance (mRMR), and combine it with an ABC algorithm, mRMR-ABC, to select informative genes from microarray profile. The new approach is based on a support vector machine (SVM) algorithm to measure the classification accuracy for selected genes. We evaluate the performance of the proposed mRMR-ABC algorithm by conducting extensive experiments on six binary and multiclass gene expression microarray datasets. Furthermore, we compare our proposed mRMR-ABC algorithm with previously known techniques. We reimplemented two of these techniques for the sake of a fair comparison using the same parameters. These two techniques are mRMR when combined with a genetic algorithm (mRMR-GA) and mRMR when combined with a particle swarm optimization algorithm (mRMR-PSO). The experimental results prove that the proposed mRMR-ABC algorithm achieves accurate classification performance using small number of predictive genes when tested using both datasets and compared to previously suggested methods. This shows that mRMR-ABC is a promising approach for solving gene selection and cancer classification problems.

## 1. Introduction

Microarray or gene expression profiling is applied to compare and determine the gene expression level and pattern for different cell types or tissue samples in a single experiment. Thus, we can determine the more informative genes that are responsible for causing a specific disease or cancer [1, 2]. Gene expression measures the activity degree for gene in a given tissue of the body. Individual genes can be active (switch on) or inactive (switch off) depending on the needs and circumstances of the body cells under particular condition [3]. Therefore, any abnormalities of gene expression level may cause the death of cells, or uncontrolled growth, as in cancer [3, 4].

However, microarray dataset suffers from the curse of dimensionality, the limited number of samples, and the irrelevant and noise genes, all of which make the classification task for a given sample more challenging [1, 5, 6]. Gene selection process aims to select the minimum number of relative and meaningful genes that are more predictive in classification process. This maximizes the classifier's ability to classify samples accurately. Lin et al. in [7] proved that the feature selection is effective and comprehensive and can significantly improve the overall classification performance. The optimal feature selection problem is considered as NP-hard problem [8]. Therefore, it is better to use heuristic approaches such as bioinspired evolutionary algorithms in order to solve this problem.

The artificial bee colony algorithm that is innovated in 2005 by Karaboga [9] is one of the bioinspired evolutionary techniques, which has been employed to identify an optimal solution in different optimization problems. ABC is inspired by the bees behaviour when looking for a good food (honey) source. In the current literature, there are many comparative studies for the performance of ABC algorithms with other bioinspired evolutionary algorithms [10–12], and the experimental results proved that the ABC algorithm is competitive. In addition, ABC has been used to solve many numerical optimization problems because it is considered to be simple technique and easy to implement and has few number of parameters. Therefore, in this paper, we propose the application of the ABC algorithm to select the predictive and informative genes from microarray gene expression profile.

Bioinspired evolutionary techniques are more applicable and accurate than the wrapper gene selection method [13] because they have the ability for searching and fining the optimal or near-optimal solutions on high-dimensional solution spaces. Furthermore, they allow searching the solution space by considering more than one attribute at the same time [13]. But, as other evolutionary approaches, the ABC has some challenging issues, especially in computational efficiency, when it is applied to complex and high-dimensional data such as microarray datasets. Therefore, to improve the performance of the ABC algorithm in high-dimensional datasets, we propose adding a feature selection algorithm, minimum redundancy maximum relevance (mRMR), as a preprocessing stage. We combine it with the ABC algorithm, mRMR-ABC, in order to select informative genes from cancer microarray profiles. This hybrid gene selection offers a good balance between filters and wrapper gene selection methods, being more computationally effective, as in filter methods, and model feature dependencies as in wrapper methods [13].

In this paper, we measure the efficiency of gene selection techniques using a support vector machine (SVM) as a classifier. An SVM displayed substantial benefits when compared to other classification approaches [14]. It is difficult to find a linear classifier to separate different classes in the dataset. An SVM solves this difficulty by mapping and converting the input space into a high-dimensional space; after that it finds a linear classification model to classify the input data with a maximum margin hyperplane. Comparing with other machine learning classification methods, SVM is more effective in high-dimensional space [15].

In the literature, there are several algorithms for gene selection and cancer classification that use a microarray. However, to our knowledge, this is the first attempt at applying ABC-based algorithm as a gene selection method for cancer classification problems using a microarray gene expression profile. The proposed algorithm is tested using six binary and multiclass gene expression microarray datasets and is also compared with original ABC, mRMR when combined with a genetic algorithm (mRMR-GA), and mRMR with a particle swarm optimization (mRMR-PSO) algorithm. In addition, we compared it with other related algorithms that have been published recently. The experimental results show improvements in both the number of selected informative genes and cancer classification accuracy.

The rest of this paper is organized as follows.  Section 2 provides a brief description of the different approaches used in our work, namely, ABC, mRMR, and SVM algorithms. We also propose both algorithms when applied to gene selection in microarray data profile. The proposed hybrid mRMR-ABC algorithm is explained in Section 3.  Section 4 outlines the experimental setup and provides results. Finally, Section 5 concludes our paper.

## 2. Methods

In this section, first we briefly introduce both artificial bee colony (ABC) and minimum redundancy maximum relevance (mRMR) filter algorithms and show how we can apply each of them for gene selection in microarray gene expression profiles. Then, general background about support vector machine (SVM) is presented.

### 2.1. Artificial Bee Colony (ABC) Algorithm for Microarray Gene Expression Profile

The ABC is a recent swarm intelligence algorithm that was invented in 2005 by Karaboga. It was inspired by the social life of bees and is used to solve the optimization problems [9]. The ABC algorithm has been widely applied in different optimization problems such as protein tertiary structures [12] and artificial neural networks [16]. To the best of our knowledge, the ABC algorithm has not been applied before to select the predictive and informative genes from cancer microarray gene profiles. The ABC algorithm is a metaheuristic evolutionary algorithm that simulates the search for food in a group of bees. The bees can be distributed at different distances (some of them quite far) in order to utilize the food resources [16]. The ABC model consists of three main components:* food sources*,* employed bees*, and* unemployed bees* [10]. All concepts are defined below along with the proposed variations when applied for gene selection in microarray data analysis.

*(i) Food Sources.* The* forager bee* measures the quality of several food sources to select the best food sources. The quality of food sources is evaluated by the distance to the hive, energy, nectar taste, and the simplicity or difficulty of the energy extracting. 

*(ii) Employed Bees.* The honeybees, or the* employed bees*, who found the food source, are equal to the number of food sources. An employed bee is exploiting a specific food source and takes the information about this food source. Subsequently, she shares the information such as distance, the direction, and the quality of the food source with other bees waiting in the hive. If the food source is exhausted, then the employed bee is considered a scout bee. 

*(iii) Unemployed Bees.* A bee that does not exploit a food source yet is called an* unemployed bee*. There are two different types of unemployed bees: scouts, who conduct random searches of the environment, and onlookers who stay in in the nest waiting the information shared by the employed bee. The most important process for knowledge collection in ABC algorithm is exchange of information between employed and unemployed bees.

**Figure 1:**
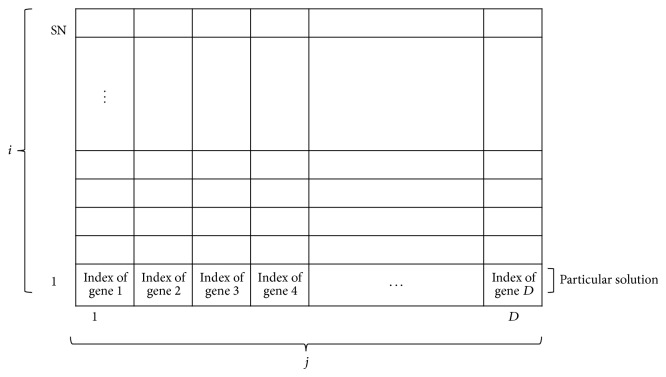
The representation of solution space (foods) for the ABC algorithm (when applied on microarray dataset). SN is the number of food sources, which represent the solutions containing indices of genes in a microarray gene expression profile, and *D* represents the number of informative genes to be optimized for each solution. Each cell represents different genes indices.

In this paper, we made some changes to the ABC algorithm representation in order to use it to solve the microarray gene selection problem. The representation of solution space (foods) for the ABC algorithm when applied on a microarray dataset is illustrated in Figure 1. The ABC algorithm first produces an initial and random solution of size SN, where SN refers to the total number of food sources. When applying an ABC algorithm to gene selection for microarray data analysis, as illustrated in Figure 1, each solution represents a group of numbers. These numbers are the indices of genes in the microarray gene expression profile (i.e., the position of food source). This is shown as *x*_*ij*_, where *i* represents a particular solution (*i* = 1,2,…, SN), and each solution is a *D*-dimensional vector (*j* = 1,2, 3,…, *D*), where *D* represents the number of informative genes to be optimized in each solution. Each cell, *x*_*ij*_, represents the corresponding gene index.

After initialization of random solutions (populations), the ABC algorithm starts searching for the optimal solution. In the ABC algorithm, each cycle of the search consists of three phases: (1)* the employed bees phase:* in which employed bees are sent to their food sources to evaluate the amount of nectar contained in each source; (2)* the onlookers phase*: in which, after receiving the nectar information for the food sources, the onlookers select the food source regions and evaluate the amount of nectar in the food sources; (3)* the scouts bee phase*: in which the scout bees are designated as such. The first half of the colony includes the employed bees, while the onlookers is considered as the second half. Each food source has only one employed bee. During searching in the solution space, the onlookers and employed bees deal with the exploitation process, while the scouts bees focus on the exploration process. In the next subsections, we will describe each of the phases and how can we apply them for microarray gene expression analysis.

#### 2.1.1. Employed Bee Phase

In this phase, the employee bees search around the solutions (food resources) at *x*_*i*_ and will search for the better genes index at the new location *v*_*i*_. Identification of the new gene index takes place by the following equation [17]:

(1) $$\begin{array}{c} v_{ij}=x_{ij}+R_{ij}\left(x_{ij}-x_{kj}\right), \end{array}$$

where *v*_*i*_ = [*v*_*i*1_, *v*_*i*2_,…, *v*_*in*_] is the new gene indices (location vector of the bees), *x*_*i*_ = [*x*_*i*1_, *x*_*i*2_,…, *x*_*in*_] is the current gene indices (location vector of the *i*th bee), *k* (*k* ≠ *j*) is a correct random number in [1, SN], and the SN is the number of the solutions (artificial bees). *R*_*ij*_ is a random number uniformly distributed in [−1,1]. The random *x*_*ij*_ numbers selection from the microarray gene index is done by the following equation [17]:

(2) $$\begin{array}{c} x_{ij}=L_{j}+\text{rand}\left(0,1\right)\times \left(U_{j}-L_{j}\right), \end{array}$$

where *U*_*j*_ and *L*_*j*_ are the top limit and the down limit of the *x*_*i*_ variable, respectively, *U*_*j*_ = (Maximum_gene_index − 1), and *L*_*j*_ = 0 while rand( ) is the random numbers function in (0,1). When the new index of the gene is identified, the optimization of it must be calculated based on the fitness function. In our problem, the fitness value fit_*i*_ is determined according to the solution classification accuracy using an SVM classifier. When the new fitness value is better than the old fitness values, then the bee changes its solution to the new solution; otherwise it stays in its solution.

#### 2.1.2. Onlooker Bee Phase

After all employed bees complete the searching for the best solutions, the information is shared with onlooker bees. An onlooker bee selects the genes depending on their highest probability value, as roulette wheel selection strategy in genetic algorithm (GA) as follows: the possibility *P*_*i*_ of selecting the particular solution (food source) by the onlooker bees is calculated using the following equation:

(3) $$\begin{array}{c} P_{i}=\frac{{\text{fit}}_{i}}{\sum_{j=1}^{\text{SN}}{\text{fit}}_{i}}. \end{array}$$

#### 2.1.3. Scout Bee Phase

Every bee (employee or onlooker) is looking for predictive genes for a specific and limited number of cycles; when the fitness value does not improve, the employee bee becomes a scout bee. A solution which could not be improved for (limit) trials becomes a scout bee. A scout bee randomly generates an index of genes in the solutions search space.

It is worth mentioning that the ABC algorithm faces some challenging problems, especially in computational efficiency, when it is applied on complex and high-dimensional data such as a microarray dataset. This motivates us to solve these difficulties and further improve the performance of the ABC algorithm by proposing a hybrid technique between the ABC algorithm and mRMR filter gene selection approach, namely, the mRMR-ABC algorithm. In the following subsection, we explain the mRMR algorithm when applied to our problem.

### 2.2. Minimum Redundancy Maximum Relevance (mRMR) Filter Algorithm

The gene selection process is very impotent for accurate classification prediction and the mRMR method can significantly improve the classification accuracy [18]. In a high-dimensional microarray dataset, because there are thousands of genes, it is inefficient to adopt an evolutionary algorithm such an artificial bee colony directly in a microarray dataset. In addition, it is difficult for a classifier to be trained accurately. Alternative techniques should be effectively adopted to solve this difficulty. Therefore, as a first step, mRMR is employed to reduce noisy and irrelevant genes. The mRMR approach was proposed by Peng et al. in 2005 [19]. It is a heuristic technique can be used for continuous and discrete datasets in order to measure the relevancy and redundancy of features and determine the promising features. In this paper, the authors perform a comparative study of mRMR with maximum relevant technique (MaxRel) and employed theme with different machine learning classifiers using four different microarray datasets (handwritten digits, arrhythmia (irregular heart beat), NCI, and lymphoma cancer). The experimental results indicate that mRMR is an effective method to improve the performance feature selection. Features selected by mRMR are more predictive and achieve accurate classification result than those selected by MaxRel.

Furthermore, Ding and Peng [18] applied mRMR feature selection method for microarray gene expression profiling. Genes selected using mRMR achieve a more coverage balance in the solution space, and they significantly improved the classification accuracy as well. The experimental study on five gene expression datasets and using four classification methods shows that the classification performance is consistently improved. In addition, Liu et al. [20] proposed an approach that adopts a mRMR filter method using mutual information operation with SVM-RFE to reduce the redundancy in the selected genes. Experimental results, based on four-benchmark cancer microarray datasets, show that the mRMR filter method is more effective when it is applied on SVM-RFE. It is also shown that mRMR can be effectively combined with other feature selectors, such as wrappers. This can be done to identify a very compact subset from candidate features at lower expense. Amine et al. [21] reported a novel gene selection method based on a hybrid approach combining an mRMR filter method with a GA wrapper method. In this study, the authors conducted a comparative study for mRMR-GA, mRMR, and GA when they applied with SVM as classifier on five different binary and multiclass cancer microarray datasets. The results from this study demonstrated the effectiveness of the integration of mRMR and GA, and it was concluded that the mRMR-GA method achieved better performance when compared to the mRMR filter and GASVM wrapper algorithms in all datasets. Meanwhile, with the same number of selected genes in this experimental result, the gene set obtained by the mRMR-GA selection was more representative of the specific class. Recently, Abdi et al. [22] introduced a novel hybrid gene selection method based on partial swarm optimization (PSO) algorithm and mRMR filter method. The numerical experimental results for colon cancer and leukemia microarray datasets prove that the mRMR-PSO algorithm achieves better classification accuracy than previously reported algorithms.

In addition, the mRMR was the successfully applied in other bioinformatics problems. For instance, Yun et al. [23] explored the effectiveness of mRMR method with GA and PSO algorithms in selecting predictive features in audiology and dermatology datasets. They concluded that using mRMR method produces better performance in terms of the classification accuracy and the relevancy of selected features. Moreover, Huang et al. [24] successfully applied the mRMR feature selection method to choose an appropriate subset of informative and relative features that are important for detection of deleterious SNPs.

**Figure 2:**
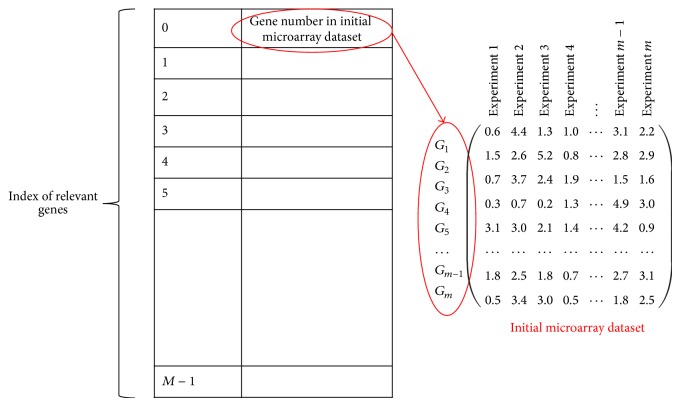
mRMR dataset that contains the gene number that is selected by the mRMR filter approach, where gene numbers are ordered by their relevancy.

In our problem, we will use mRMR gene selection method to identify the predictive genes that have minimum redundancy with other genes in microarray dataset and maximum relevancy for specific cancer classes. Thus, the mRMR method used two mutual information MI operations: one between cancer classes and each gene in order to measure the relevancy, while the second mutual information between every two genes to calculate the redundancy.  Figure 2 presents the mRMR dataset, which contains the ordered selected genes indices. The first row represents the maximum relevant and the minimum redundant genes.

*S* denotes the selected genes and Rl measures the relevancy of a group of selected genes *S* that can be defined as follows:

(4) $$\begin{array}{c} \text{Rl}=\frac{1}{\left|S\right|}\underset{G_{x}\in S}{\sum }I\left(G_{x},C\right), \end{array}$$

where *I*(*G*_*x*_, *C*) represents the value of mutual information between an individual gene *G*_*x*_ that belongs to *S* and the cancer class *C* = {*c*_1_, *c*_2_}, where *c*_1_ and *c*_2_ denote the normal and tumor classes.

When the selected genes have the maximum relevance Rl value, it is possible to have high dependency (i.e., redundancy) between these genes. Hence, the redundancy Rd of a group of selected genes *S* is defined as

(5) $$\begin{array}{c} \text{Rd}=\frac{1}{{\left|S\right|}^{2}}\underset{G_{x},G_{y}\in S}{\sum }I\left(G_{x},G_{y}\right), \end{array}$$

where *I*(*G*_*x*_, *G*_*y*_) is the mutual information between the *x*th and *y*th genes that measures the mutual dependency of these two genes.

The main purpose of applying the mRMR gene selection method is to find a subset of genes from *S* with *m* genes, {*x*_*i*_}, that either jointly have the largest dependency on the target class *c* or have the minimal redundancy on the selected gene subset *S*. Thus, Peng et al. [19] recommend searching for balanced solutions through the composite objective. This criterion combines the two criteria, which are maximal relevance criterion and minimal redundancy criterion, as follows:

(6) $$\begin{array}{c} \max\left(\text{Rl},\text{Rd}\right)=\text{Rl}-\text{Rd}. \end{array}$$

Our goal is to increase the prediction accuracy and reduce the number of selected genes. Hence, we applied the mRMR method as a preprocessing step to the ABC algorithm to improve the speed and performance of the search.

### 2.3. Support Vector Machine (SVM)

SVM algorithm is a machine learning approach based on statistical learning theory, which is proposed in 1998 by Vapnik [25]. An SVM is powerful classification algorithm that showed a good performance in a variety of bioinformatics classification tasks. In addition, SVMs are very effective classification techniques for microarray data and they significantly improved the classification accuracy performance. One of the main advantages of SVM models in cancer classification using high-dimensional data such as microarray datasets is that being able to be adjusted with all genes and at the same time with stable performance when using the full set of genes [26–29]. Its aim is to find the hyperplane that is separating the feature with the largest margin (distance between itself and the closest samples from each classes). Generally, the better SVM classifier seeks to balance between increasing the margin and reducing the number of errors. In our recent comparative study [14], we showed that machine learning classification methods produce accurate result with minimum number of genes. There are many machine learning techniques that have been applied for classifying microarray dataset, including SVM, K nearest neighbor (KNN), random forest (RF), artificial neural network (ANN), and naive Bayes (NB). Therefore, we compared the classification performance achieved by each machine learning classification algorithm that was proposed in the state-of-the-art for cancer microarray datasets, as shown in Table 1. The table also compares the classification performances for four microarray datasets. The best performance of each cancer classification approach for each microarray dataset is indicated in bold. Up to the available literature review, SVM has superior classification accuracy performance when applied on microarray data.

From early stage of the SVM, researchers have applied the linear, polynomial, and RBF kernels for classification problems [30]. It is worth pointing out that the polynomial and RBF are the nonlinear kernel, and cancer classification using microarray dataset is a nonlinear classification task [30]. Nahar et al. [30] observed from their experiment out of nine microarray datasets that the polynomial kernel is the best choice for classifying microarray datasets. Therefore, we used polynomial kernel for SVM classifier. In addition, we apply leave-one-out cross validation (LOOCV) [31] in order to evaluate the performance of our proposed algorithm and the existing methods in the literature. LOOCV is very suitable to our problem because it has the ability to prevent the “overfitting” problem [31]. In LOOCV, one sample from the original dataset is considered testing dataset, and the remaining samples are considered training dataset. This is repeated such that each sample in the microarray dataset is used once as the testing dataset.

## 3. Proposed mRMR-ABC Algorithm

**Figure 3:**
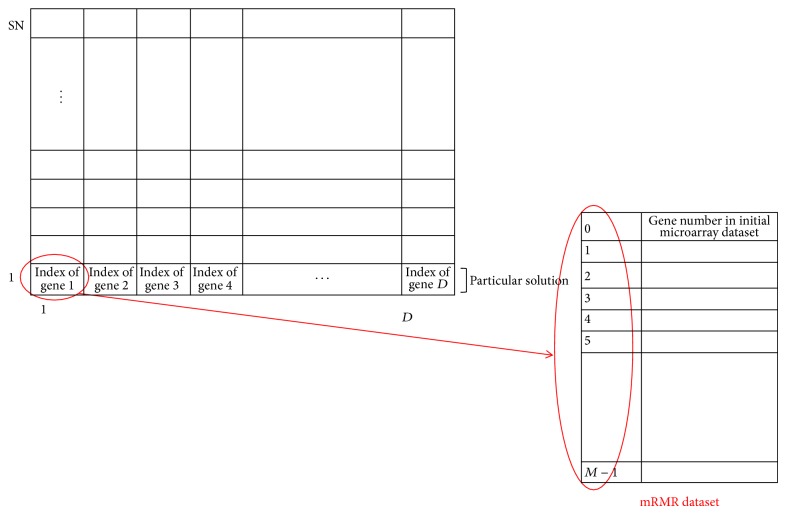
The representation of food for the proposed mRMR-ABC algorithm. Each row of food matrix represents a particular solution, which contains *D* genes indices that are to be optimized. The number of rows of food matrix equals the food number “SN”.

In this section, we introduce the proposed mRMR-ABC algorithm to select the predictive genes from the cancer microarray gene expression profile. The aim of this algorithm is to select the more informative gene in order to improve the SVM classifier accuracy performance by preselecting the relative and informative genes using the mRMR method and then estimating the best predictive genes by applying the ABC algorithm as a wrapper gene selection technique with the SVM classifier. In Figure 3, we demonstrated the solution representation for the proposed algorithm. The food sources represent the population of solutions. Each row of foods matrices is a particular solution holding *D* genes indices that are to be optimized and selected for an mRMR dataset, shown in Figure 2. It is clear that, in our proposed mRMR-ABC algorithm, we select the genes form a small dataset (mRMR dataset) that contains the informative genes. Consequently, the optimization process will be improved, compared with the original ABC algorithm that selected the genes directly for the initial microarray dataset.

**Figure 4:**
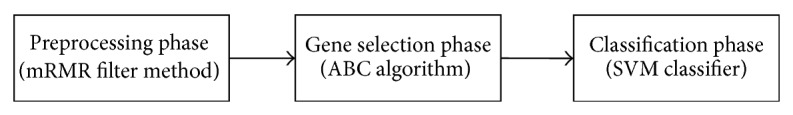
The phases of the mRMR-ABC algorithm.

As illustrated in Figure 4, our proposed algorithm consists of three phases:* preprocessing phase*,* gene selection phase*, and* classification phase*.

**Figure 5:**
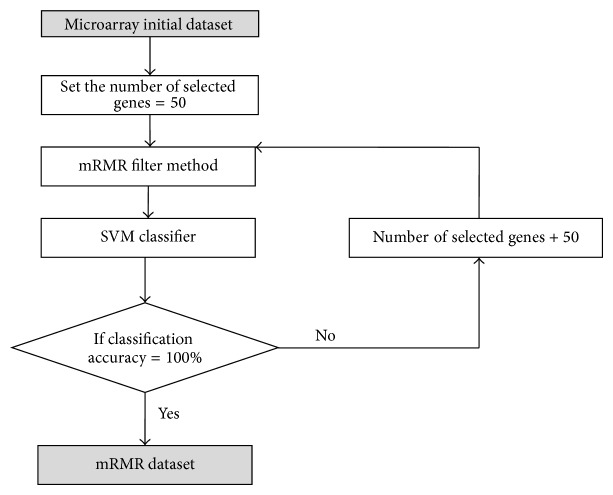
The preprocessing phase (mRMR algorithm).

*(i) Preprocessing Phase (Figure 5)*. The initial microarray gene expression profiling is filtered and preprocessed using the mRMR gene selection method. Each gene is evaluated and sorted using the mRMR mutual information MI operations as explained in Section 2.2. The highest relevant genes that give 100% classification accuracy with an SVM classifier are identified to form a new subset named the mRMR dataset, as shown in Figure 2. The mRMR dataset denotes the more relative and less redundant genes as selected by the mRMR approach. The mRMR is applied in order to filter irrelevant and noisy genes and reduces the computational load for the ABC algorithm and SVM classifier. 

**Figure 6:**
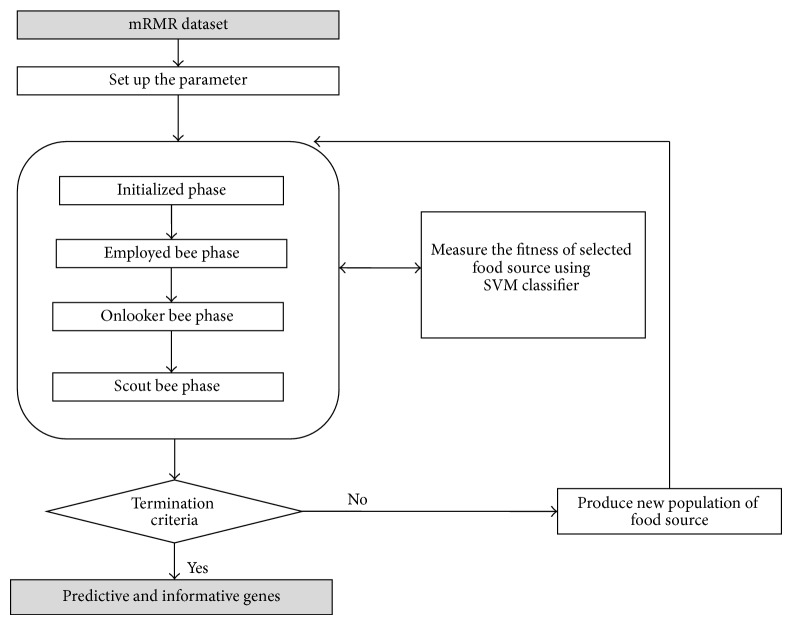
The gene selection phase (ABC algorithm).

*(ii) Gene Selection Phase (Figure 6).* An ABC algorithm is developed as described in Section 2.1 to select the most informative and predictive genes from an mRMR dataset that give the highest classification accuracy with an SVM classifier. Figure 3 illustrates the representation of the food source or the solution space for the proposed mRMR-ABC algorithm. Each solution is represented as a group of genes indices that are selected form the mRMR dataset. In a gene selection problem, each solution (i.e., subset of selected genes) is associated with the fitness value, which is the classification accuracy using an SVM classifier. 

**Figure 7:**
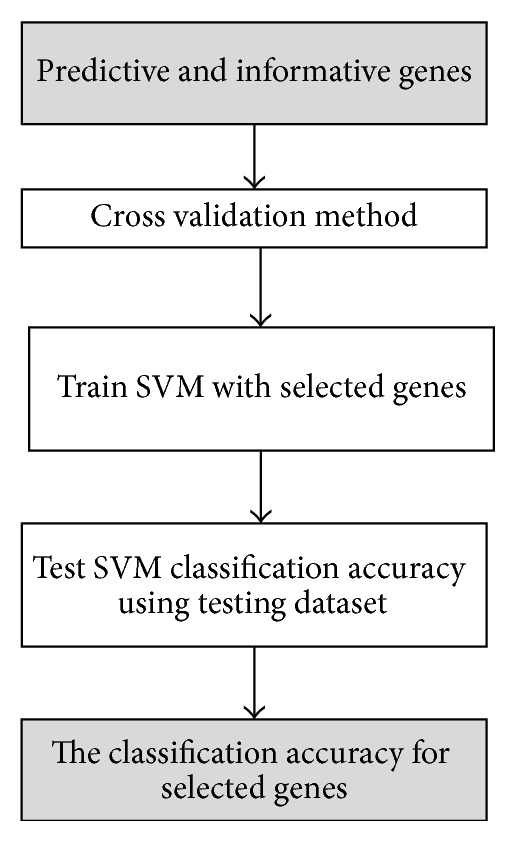
The classification phase (SVM classifier).

*(iii) Classification Phase (Figure 7).* Use the informative and predictive genes that are generated from the ABC algorithm in the second phase to train the SVM classifier. The SVM is applied again to classify the testing microarray dataset and restore the classification accuracy.

The main steps for proposed algorithm (mRMR-ABC) are presented as follows.

 (1) Preprocess microarray dataset using* mRMR* filtering method.
 (2) Initialize population.
 
*Repeat*
 (3) Place the employed bees on their food sources.
 (4) Place the onlooker bees on the food sources depending on their nectar amounts.
 (5) Send the scouts to the search area for discovering new food sources.
 (6) Memorize the best food source found so far
 
*Until requirements are met*
 (7) Classify microarray dataset using SVM classifier.

In addition, the pseudocode for the proposed mRMR-ABC algorithm is presented as follows.

1. Select the high relevant genes subset using mRMR filter method that gives 100% classification accuracy with SVM classifier.
2. Set the parameter: Max cycles, colony size, and limit.
3. Initialize the food sources.
4. Evaluate the food sources by calculating the fitness, which is the classification accuracy using SVM classifier.
5. *Cycle* ← 1.
6. **While** *Cycle* < *MaxCycles* **Do**.
7. Generate new solutions using employed bees.
8. Evaluate the new solutions by calculating the fitness (SVM classification accuracy).
9. Apply greedy selection operation.
10. Calculate the probability values using fitness function.
11. Generate new solutions using onlooker bees based on the probability of food source.
12. Evaluate the new solutions by calculating the fitness (SVM classification accuracy).
13. Apply greedy selection operation.
14. Determine abandoned solutions and generate new solutions randomly using scouts.
15. Memorize the best solution found so far.
16. *Cycle* ← *Cycle* + 1.
17. **End While**.
18. Return a best solution (predictive and informative genes).
19. Train the SVM classifier using selected genes.
20. Classify microarray dataset using SVM.
21. Return the classification accuracy.

## 4. Experimental Setup and Results 

### 4.1. Experiential Setup

In this section, we evaluate the overall performance of gene selection methods using six popular binary and multiclass microarray cancer datasets, which were downloaded from http://www.gems-system.org/. These datasets have been widely used to benchmark the performance of gene selection methods in bioinformatics field. The binary-class microarray datasets are* colon* [32],* leukemia* [32, 33], and* lung* [34] while the multiclass microarray datasets are* SRBCT* [35],* lymphoma* [36], and* leukemia* [37]. In Table 2, we present a detailed description of these six benchmark microarray gene expression datasets with respect to the number of classes, number of samples, number of genes, and a brief description of each dataset construction.

Table 3 shows the control parameters for the mRMR-ABC algorithm that was used in our experiments. The first control parameter is the* bee colony size* or population, with a value of 80. The second control parameter is the* maximum cycle*, which is equal to the maximum number of generations. A value of 100 is used for this parameter. Another control parameter is the* number of runs*, which was used as stopping criterion, and we used a value of 30 in our experiments, which has been shown to be acceptable. The last control parameter is the* limit*, which represents the maximum number of iterations allowed when the food source is not improved (exhausted). If the food source exceeds this limit, it will be selected by the scout bee. A value of 5 iterations is used for this parameter.

In this study, we tested the performance of the proposed mRMR-ABC algorithm by comparing it with other standard bioinspired algorithms, including ABC, GA, and PSO. We compared the performance of each gene selection approach based on two parameters: the classification accuracy and the number of predictive genes that have been used for cancer classification. Classification accuracy is the overall correctness of the classifier and is calculated as the sum of correct cancer classifications divided by the total number of classifications. It is computed by the expression shown below:

(7) $$\begin{array}{c} \text{Classification}\_\text{Accuracy}=\frac{\text{CC}}{N}\times 100, \end{array}$$

where *N* is the total number of the instances in the initial microarray dataset. And, CC refers to correctly classified instances.

We apply leave-one-out cross validation (LOOCV) [31] in order to evaluate the performance of our proposed algorithm and the existing methods in the literature. LOOCV is very suitable to our problem because it has the ability to prevent the “overfitting” problem [31]. It also provides an unbiased estimate of the generalization error for stable classifiers such as the SVM classifier. In LOOCV, one sample from the original dataset is considered testing dataset, and the remaining samples are considered training dataset. This is repeated such that each sample in the microarray dataset is used once as the testing dataset. We implement GA, PSO algorithm, and SVM using the Waikato Environment for Knowledge Analysis (WEKA version 3.6.10), an open source data mining tool [38]. Furthermore, in order to make experiments more statistically valid, we conduct each experiment 30 times on each dataset. In addition, best, worst, and average results of the classification accuracies of the 30 independent runs are calculated in order to evaluate the performance of our proposed algorithm.

### 4.2. Experimental Results

**Figure 8:**
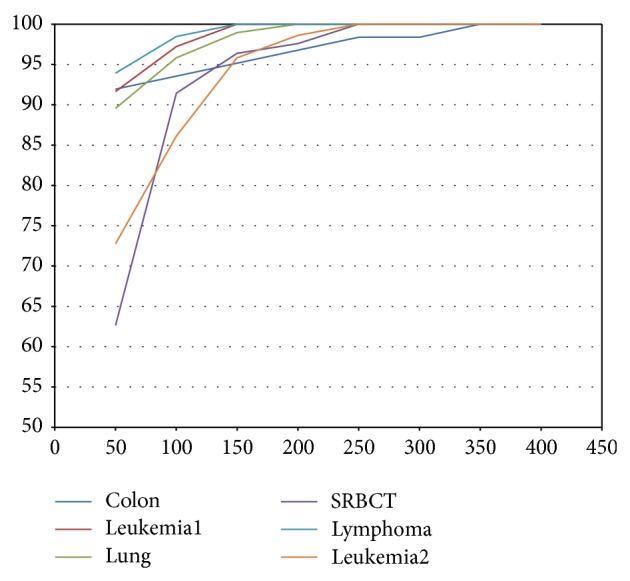
The classification accuracy performance of the mRMR method with an SVM classifier for all microarray datasets.

In this section, we present and analyze the results that are obtained by our algorithm. As a first step, we employed the mRMR method to identify the top relevant genes that give 100% accuracy with an SVM classifier. From Table 4 and Figure 8, we can see that the top 150 genes in the leukemia1 dataset generate 100% classification accuracy while in the colon dataset, we can get 100% accuracy using 350 genes. For the lung dataset, we achieved 100% accuracy using 200 genes and 250 genes to get the same classification accuracy for the SRBCT dataset. In addition, using 150 high relevant genes from the lymphoma dataset and 250 genes from the leukemia2 dataset, we achieved 100% classification accuracy. Then we used these high relevant genes as input in the ABC algorithm to determine the most predictive and informative genes.

We compared the performance of the proposed mRMR-ABC algorithm with the original ABC algorithm, when using SVM as a classifier with the same number of selected genes for all six benchmark microarray datasets. The comparison results for the binary-class microarray datasets: colon, leukemia1, and lung are shown in Tables 5, 6, and 7, respectively while Tables 8, 9, and 10, respectively, present the comparison result for multiclass microarray datasets: SRBCT, lymphoma, and leukemia2. From these tables, it is clear that our proposed mRMR-ABC algorithm performs better than the original ABC algorithm in every single case (i.e., all datasets using a different number of selected genes).

In this research, we reimplement mRMR with particle swarm optimization (mRMR-PSO) and mRMR with genetic algorithm (mRMR-GA) in order to compare the performance of the mRMR-ABC algorithm with the same parameters. We also compare it with published results for recent gene selection algorithms. Notably, all these algorithms have been combined with the SVM as a classification approach.

Table 11 shows the numerical comparative results of the mRMR-ABC algorithm and related algorithms proposed in the literature. Compared with the mRMR-ABC algorithm, the* mAnt* method opposed by Yu et al. (2009) [39] selected fewer genes on the colon dataset. The* mAnt* algorithm selected 8 genes and achieved 91.5% classification accuracy. In contrast, the mRMR-ABC algorithm selects 15 genes and achieves 96.77% classification accuracy. For the leukemia1 dataset, the mRMR-ABC algorithm achieves 100% classification accuracy with 14 selected genes. In comparison, the* mRMR-PSO* algorithm proposed by Abdi et al. (2012) [22] achieved 100% classification accuracy; however, their selected genes are greater. For the lung dataset, the mRMR-ABC algorithm selected 8 genes to achieve 100% classification accuracy. The* mRMR-GA *algorithm proposed by Amine et al. (2009) [21] selected 15 genes in order to achieve 100% accuracy on the same dataset.

For SRBCT dataset, the* MLHD-GA* algorithm proposed by Huang et al. (2007) [40] achieved 100% classification accuracy using 11 selected genes. By contrast, the mRMR-ABC algorithm selects 10 genes and achieves 100% classification accuracy. Although there are many existing algorithms that achieve 100% for the lymphoma dataset, the mRMR-ABC algorithm selected a smaller number of predictive genes. The mRMR-ABC selected only five genes to achieve 100% classification accuracy for the lymphoma dataset. Finally, for the leukemia2 dataset, the mRMR-ABC method selected 20 genes to achieve 100% classification accuracy. It exceeds all the other methods in performance except the* MLHD-GA* algorithm proposed by Huang et al. (2007) [40], which selected 9 genes to achieve 100% classification accuracy.

Generally, most related algorithms aim to identify the minimum number of selected genes with high classification accuracy. In comparison, the mRMR-ABC algorithm selects fewer genes than most of the algorithms under comparison with relatively high classification accuracy. On the other hand, for the algorithms that select fewer genes than the mRMR-ABC algorithm, their classification accuracy is less than the mRMR-ABC classification accuracy.

The explanation of the best predictive and highly frequent genes that give highest classification accuracy for all microarray datasets using mRMR-ABC algorithm has been reported in Table 12.

It is worth mentioning that the accuracy of the mRMR filter method when it is combined with ABC generally outperforms the classification accuracy of ABC algorithm without mRMR. Thus, the mRMR is a promising method for identifying the relevant genes and omitting the redundant and noisy genes. We can conclude that the proposed mRMR-ABC algorithm generates accurate classification performance with minimum number of selected genes when tested using all datasets as compared to the original ABC algorithm under the same cross validation approach. Therefore, the mRMR-ABC algorithm is a promising approach for solving gene selection and cancer classification problems.

## 5. Conclusion

In this research paper, we proposed applying ABC algorithm for microarray gene expression profile. In addition, we proposed a new artificial bee colony-based algorithm called the mRMR-ABC hybrid gene selection approach to be combined with SVM as a classifier. It can be used to solve classification problems that deal with high-dimensional datasets, especially microarray gene expression profile. Up to our knowledge, the ABC algorithm has not yet been applied as a gene selection technique for a microarray dataset, so this is the first attempt. Our proposed mRMR-ABC algorithm is a three-phase method; the mRMR filter technique is adopted to identify the relative and informative gene subset from the candidate microarray dataset. Then the ABC algorithm is employed to select the predictive genes from the mRMR genes subset. Finally, the SVM classifier was trained and tested using the selected genes and returned the classification accuracy. Extensive experiments were conducted using six binary and multiclass microarray datasets. The results showed that the proposed algorithm achieves superior improvement when it is compared with the other previously proposed algorithms.

Recently, Lin et al. [41] proposed a new hybrid approach called libD3d; this approach is a hybrid model of ensemble pruning that is based on k-means clustering and the framework of dynamic selection and circulating in combination with a sequential search method. Empirical study shows that libD3C exhibits a competitive accuracy performance against other high-performance methods and verifies the feasibility of multiclass classification. Therefore, in the future, we intend to use licD3C for microarray cancer classification, which, up to our knowledge, has not been applied yet. In addition, we would like to conduct more experimental results on more real and benchmark datasets to verify and extend this proposed algorithm. Moreover, mRMR-ABC algorithm can be considered as a general framework that can be used to solve various optimization problems.

## Figures and Tables

**Table 1:** Comprehensive comparison between the state-of-the-art machine learning cancer classification methods in terms of classification accuracy and number of selected genes for the four benchmark microarray datasets (colon, leukemia, lung, and prostate). The number between parenthesis denotes the number of selected genes. The best classification performance in each gene selection approach for each microarray dataset is indicated in bold.

Cancer classification methods	Colon	Leukemia	Lung	Prostate
ANN [42]			93.43 (40)	97.33 (10)
NB [20]	88.79 (8)	100 (8)		98.04 (8)
KNN [20]	77.42 (12)	100 (12)		97.06 (12)
KNN [43]	100 (9)			
KNN [44]	100 (9)			
RF [45]	84.4 (14)	91.3 (2)		93.9 (18)
SVM [46]		94 (20)		
SVM [26]	88.41 (25)		99.63 (5)	90.26 (4)
SVM [47]	88.18 (95)	96.88 (88)	99.90 (29)	93.41 (85)
SVM [20]	91.68 (78)	98.35 (37)		**98.29 **(10)
SVM [27]	**100 **(8)	** 100 **(5)		
SVM [48]	91.67 (4)			
SVM [49]		100 (6)	98.66 (2)	
SVM [50]		98.57 (7)		
SVM [21]	95 (5)	**100 **(5)	**100 **(5)	
SVM [28]	99.41 (10)	100 (25)		

**Table 2:** Statistics of microarray cancer datasets.

Microarray datasets	Number of classes	Number of samples	Number of genes	Description
Colon [32]	2	62	2000	40 cancer samples and 22 normal samples
Leukemia1 [33]	2	72	7129	25 AML samples and 47 ALL samples
Lung [34]	2	96	7129	86 cancer samples and 10 normal samples
SRBCT [35]	4	83	2308	29 EWS samples, 18 NB samples, 11 BL samples, and 25 RMS samples
Lymphoma [36]	3	62	4026	42 DLBCL samples, 9 FL samples, and 11 B-CLL samples
Leukemia2 [37]	3	72	7129	28 AML sample, 24 ALL sample, and 20 MLL samples

**Table 3:** mRMR-ABC control parameters.

Parameter	Value
Colony size	80
Max cycle	100
Number of runs	30
Limit	5

**Table 4:** The classification accuracy performance of the mRMR method with an SVM classifier for all microarray datasets.

Number of genes	Colon	Leukemia1	Lung	SRBCT	Lymphoma	Leukemia2
50	91.94%	91.66%	89.56%	62.65%	93.93%	77.77%
100	93.55%	97.22%	95.83%	91.44%	98.48%	86.11%
150	95.16%	100%	98.95%	96.39%	100%	95.83%
200	96.77%	100%	100%	97.59%	100%	98.61%
250	98.38%	100%	100%	100%	100%	100%
300	98.38%	100%	100%	100%	100%	100%
350	100%	100%	100%	100%	100%	100%
400	100%	100%	100%	100%	100%	100%

**Table 5:** Comparison between mRMR-ABC and ABC algorithms classification performance when applied with the SVM classifier for colon dataset.

Number of genes	Classification accuracy					
	mRMR-ABC			ABC		
	Best	Mean	Worst	Best	Mean	Worst
3	88.71%	87.50%	85.48%	87.10%	85.91%	83.87%
4	90.23%	88.27%	87.10%	87.10%	86.71%	85.48%
5	91.94%	89.50%	87.10%	90.32%	87.98%	85.48%
6	91.94%	90.12%	87.10%	90.32%	88.44%	85.48%
7	993.55%	91.64%	88.81%	91.94%	90.20%	88.81%
8	93.55%	91.80%	88.81%	91.94%	90.61%	88.81%
9	93.55%	92.11%	90.16%	91.94%	90.95%	88.81%
10	93.55%	92.74%	90.16%	93.55%	91.31%	88.81%
15	96.77%	93.60%	91.93%	93.55%	91.38%	90.32%
20	96.77%	94.17%	91.93%	95.61%	92.44%	90.32%

**Table 6:** Comparison between mRMR-ABC and ABC algorithms classification performance when applied with the SVM classifier for leukemia1 dataset.

Number of genes	Classification accuracy					
	mRMR-ABC			ABC		
	Best	Mean	Worst	Best	Mean	Worst
2	91.66%	89.63%	81.94%	87.5%	86.45%	81.94%
3	93.05%	90.37%	83.33%	88.88%	89.82%	83.33%
4	94.44%	91.29%	86.11%	88.8%	91.15%	83.33%
5	95.83%	92.82%	88.88%	91.66%	91.89%	87.5%
6	95.83%	92.82%	90.32%	91.99%	92.04%	87.5%
7	97.22%	93.10%	90.32%	93.05%	92.23%	87.5%
10	98.61%	94.44%	91.66%	93.05%	92.38%	88.88%
13	98.61%	94.93%	91.66%	93.05%	92.44%	88.88%
14	100%	95.83%	93.05%	93.05%	92.51%	88.88%

**Table 7:** Comparison between mRMR-ABC and ABC algorithms classification performance when applied with the SVM classifier for lung dataset.

Number of genes	Classification accuracy					
	mRMR-ABC			ABC		
	Best	Mean	Worst	Best	Mean	Worst
2	96.87%	95.83%	93.75%	88.54%	87.5%	84.37%
3	97.91%	96.31%	93.75%	89.58%	88.54%	84.37%
4	98.95%	97.91%	96.87%	91.66%	89.58%	87.5%
5	98.95%	97.98%	96.87%	92.70%	90.03%	88.54%
6	98.95%	98.27%	96.87%	94.79%	91.66%	88.54%
7	98.95%	98.53%	96.87%	95.83%	92.18%	89.58%
8	100%	98.95%	96.87%	97.91%	93.75%	91.66%

**Table 8:** Comparison between mRMR-ABC and ABC algorithms classification performance when applied with the SVM classifier for SRBCT dataset.

Number of genes	Classification accuracy					
	mRMR-ABC			ABC		
	Best	Mean	Worst	Best	Mean	Worst
2	75.90%	71.08%	68.67%	72.28%	69.87%	67.46%
3	85.54%	79.51%	71.08%	73.34%	71.08%	68.67%
4	87.95%	84.33%	77.10%	84.33%	81.92%	77.10%
5	91.56%	86.74%	84.33%	87.95%	84.33%	77.10%
6	95.36%	91.56%	87.99%	92.77%	87.99%	84.33%
8	97.59%	94.05%	89.15%	93.97%	89.15%	84.33%
10	100%	96.30%	92.77%	95.36%	91.56%	89.15%

**Table 9:** Comparison between mRMR-ABC and ABC algorithms classification performance when applied with the SVM classifier for lymphoma dataset.

Number of genes	Classification accuracy					
	mRMR-ABC			ABC		
	Best	Mean	Worst	Best	Mean	Worst
2	86.36%	86.36%	86.36%	86.36%	86.36%	86.36%
3	93.93%	90.90%	86.36%	89.39%	87.87%	86.36%
4	96.96%	92.42%	89.39%	93.93%	89.39%	86.36%
5	100%	96.96%	93.93%	96.96%	92.42%	90.90%

**Table 10:** Comparison between mRMR-ABC and ABC algorithms classification performance when applied with the SVM classifier for Leukemia2 dataset.

Number of genes	Classification accuracy					
	mRMR-ABC			ABC		
	Best	Mean	Worst	Best	Mean	Worst
2	84.72%	84.72%	84.72%	84.72%	84.72%	84.72%
3	87.5%	86.11%	84.72%	86.11%	85.23%	84.72%
4	90.27%	87.5%	84.72%	87.5%	86.11%	84.72%
5	90.27%	88.88%	86.11%	87.5%	86.45%	84.72%
6	94.44%	90.27%	87.5%	90.27%	88.88%	86.11%
7	93.05%	89.49%	88.88%	90.27%	89.22%	86.11%
8	94.44%	91.66%	87.5%	91.66%	90.27%	88.88%
9	94.44%	92.38%	87.5%	93.05%	91.46%	88.88%
10	95.83%	91.66%	88.88%	93.05%	91.98%	88.88%
15	98.61%	94.44%	91.66%	94.44%	92.78%	90.27%
18	98.61%	95.67%	91.66%	95.83%	92.99%	90.27%
20	100%	96.12%	95.83%	97.22%	93.15%	91.66%

**Table 11:** The classification accuracy of the existing gene selection algorithms under comparison when combined with the SVM as a classifier for six microarray datasets. Numbers between parentheses denote the numbers of selected genes.

Algorithms	Colon	Leukemia1	Lung	SRBCT	Lymphoma	Leukemia2
mRMR-ABC	96.77 (15)	100 (14)	100 (8)	100 (10)	100 (5)	100 (20)
ABC	95.61 (20)	93.05 (14)	97.91 (8)	95.36 (10)	96.96 (5)	97.22 (20)
mRMR-GA	95.61 (83)	93.05 (51)	95.83 (62)	92.77 (74)	93.93 (43)	94.44 (57)
mRMR-PSO	93.55 (78)	95.83 (53)	94.79 (65)	93.97 (68)	96.96 (82)	95.83 (61)
PSO [51]	85.48 (20)	94.44 (23)				
PSO [52]	87.01 (2000)	93.06 (7129)				
mRMR-PSO [22]	90.32 (10)	100 (18)				
GADP [27]					100 (6)	
mRMR-GA [21]			100 (15)		95 (5)	
ESVM [53]			95.75 (7)	98.75 (6)		
MLHD-GA [40]			97.1 (10)	100 (11)	100 (6)	100 (9)
CFS-IBPSO [50]					100 (6)	98.57 (41)
GA [54]	93.55 (12)					
mAnt [39]	91.5 (8)				100 (7)	

**Table 12:** The best predictive genes that give highest classification accuracy for all microarray datasets using mRMR-ABC algorithm.

Datasets	Predictive genes	Accuracy
Colon	Gene115, Gene161, Gene57, Gene70, Gene12, Gene132, Gene84, Gene62, Gene26, Gene155, Gene39, Gene14, Gene1924, Gene148, and Gene21	96.77%

Leukemia1	M31994_at, U07563_cds1_at, Y07604_at, J03925_at, X03484_at, U43522_at, U12622_at, L77864_at, HG3707-HT3922_f_at, D49950_at, HG4011-HT4804_s_at, Y07755_at, M81830_at, and U03090_at	100%

Lung	U77827_at, D49728_at, HG3976-HT4246_at, X77588_s_at, M21535_at, L29433_at, U60115_at, and M14764_at	100%

SRBCT	Gene795, Gene575, Gene423, Gene2025, Gene1090, Gene1611, Gene1389, Gene338, Gene1, and Gene715	100%

Lymphoma	Gene1219X, Gene656X, Gene2075X, Gene3344X, and Gene345X	100%

Leukemia2	Y09615_atD87683_at, U31973_s_at, U68031_at, V00571_rna1_at, L39009_at, U37529_at, U35407_at, X93511_s_at, L15533_rna1_at, X00695_s_at, H46990_at, U47686_s_at, L27624_s_at, S76473_s_at, X16281_at, M37981_at, M89957_at, L05597_at, and X07696_at	100%
